# A predictive model to estimate fever after receipt of the second dose of Pfizer‐BioNTech coronavirus disease 2019 vaccine: An observational cohort study

**DOI:** 10.1002/hsr2.742

**Published:** 2022-07-20

**Authors:** Satoru Chiba, Kaoru Shinohara

**Affiliations:** ^1^ Department of Internal Medicine, Sapporo Suzuki Hospital Medical Corporation Kenseikai Hokkaido Japan; ^2^ Department of Psychiatric Medicine, Sapporo Suzuki Hospital Medical Corporation Kenseikai Hokkaido Japan

**Keywords:** body surface area, body weight, perennial allergic rhinitis, postvaccination fever, serum hemoglobin concentration

## Abstract

**Background and Aims:**

Fever after coronavirus disease 2019 (COVID‐19) vaccination is generally a mild and benign event, but can cause excessive anxiety in younger adults. This study aimed to find key factors that include allergic diseases or physique that determine fever after vaccination.

**Methods:**

We conducted an observational cohort study in our hospital to assess post‐COVID‐19 vaccination fever from April to June 2021. A total of 153 medical personnel aged 22–86 years of age were involved in the study to receive two doses, intramuscularly 21 days apart, of the Pfizer‐BioNTech COVID‐19 vaccine (30 μg per dose). Vaccination records were taken more than 72 h after vaccination. Clinical and laboratory variables (age, sex, allergy history, weight, height, serum hemoglobin concentration, and these derivatives) were examined by multivariable logistic regression analysis using the peak axillary temperature in the 4‐day period after the second vaccination as a dependent variable.

**Results:**

No serious safety problems were detected. The incidence of a postsecond vaccination fever of 37.3°C or above was 29.4%. Logistic regression analysis found age, history of perennial allergic rhinitis, body surface area, body weight, percent overweight, and serum hemoglobin concentration as independent predictors of postvaccination fever. The characteristics of this individual were incorporated into the numerical model of human thermoregulation. The evaluation of this model had a sensitivity of 66.1% and a specificity of 90.7% in the detection of postvaccination fever. The multiple coefficient of determination (*R* ^2^) was 0.410.

**Conclusion:**

The COVID‐19 vaccine induced higher rates of fever during the 4‐day period after the second vaccination. Younger age, part of the allergy history, small and light body, and concentrated blood were associated with postvaccination fever.

## 1 INTRODUCTION

Coronavirus disease 2019 (COVID‐19) was declared a pandemic by the World Health Organization on March 11, 2020,^1^ and the pandemic continues to worsen throughout the world. Elderly patients with underlying diseases and medical personnel are placed in a greater risk situation for COVID‐19.^2^ Although many drugs have been repurposed in the treatment of COVID‐19, they remain supportive therapeutic options.^3^, ^4^, ^5^ Safe and effective vaccines are urgently needed to control the COVID‐19 pandemic. Two 30 μg doses of the Pfizer‐BioNTech's mRNA COVID‐19 vaccine (BNT162b2) were shown to improve an immune response with a high level of severe acute respiratory syndrome coronavirus‐2 (SARS‐CoV‐2) neutralizing antibodies and the reactogenicity profile of BNT162b2 showed transient local and systemic reactions.^6^, ^7^ Furthermore, achieving high vaccination coverage is critical to reduce COVID‐19 associated mortality.

Fever can occur after every vaccination and has not been observed much in youth after COVID‐19 vaccination.^7^ Because vaccination‐associated fever and anaphylaxis can cause young people to avoid COVID‐19 vaccination, it is necessary to understand how individual factors impact fever. No study clearly showed that fever after COVID‐19 vaccination is related to underlying allergic diseases and physique. In the present study, we used a multivariable statistical approach to identify an individualized model of human thermoregulation for stimulation of vaccination in adults. This study aimed to find key factors that include allergic diseases or physique that determine fever after vaccination.

## 2 METHODS

### 2.1 Study design and participants

A total of 163 medical personnel from our hospital were prospectively recruited in this observational cohort study from April 16 to June 23, 2021. One hundred and fifty‐three eligible participants 22–86 years of age who were healthy or had stable chronic illnesses were assigned 30 μg of BNT162b2 (0.3 ml volume per dose), and given two doses, 21 days apart, injected into the deltoid muscle. The vaccination staff responsible for the safety evaluation observed people with a history of immediate allergic reactions to a vaccine or other drugs or any history of anaphylaxis for 30 min after vaccination. All other participants were observed for 15 min after vaccination for any acute reactions. Body temperature was measured at the axilla using the conventional digital thermometer. Because postvaccination fever generally lasts about 48 h,^7^ fever response was observed at least 72 h after vaccination. Each participant was instructed to record the temperature each day on a prospective diary card over 3 days after vaccination. Serious adverse events were recorded throughout the study period. Data on history and physical examination were compiled using a predesigned format. The percent overweight was determined using the following formula: ([actual body weight − standard weight]/Standard weight) × 100 (%).^8^ Blood samples were taken from participants during their health check between June and September 2021. The clinical hematology laboratory performed a serum hemoglobin concentration assessment using the cyanmethemoglobin method (Bio Medical Laboratories).^9^

### 2.2 Definition of fever

The axillary temperature is used in routine clinical practice due to ease of use and safety, although the gold standard definition of a fever is a rectal temperature of 38°C or higher.^10^ In this study, postvaccination fever was defined as an axillary temperature of 37.3°C or higher within the first 72 h of vaccination, because the mean difference in temperature (rectal minus axillary temperature) for the conventional digital thermometer was 0.85°C.^11^ I advised the participants not to administer acetaminophen below their temperature of 37.3°C.

### 2.3 Statistical analysis

The results were examined by univariable and multivariable analysis. The variables entered into the multivariable logistic regression analysis were those with perceived clinical relevance, those identified by the univariable analysis in this study, or those reported as diagnostic value by other study.^12^ Microsoft Excel.2010 was used for statistical calculations. Data are presented as mean (standard deviation) or percentages of participants. *p* < 0.05 was considered significant for all tests.

The effect size (Cohen's *d*) was calculated to quantify the size of differences between two groups, with values of 0.2–<0.5 considered as small, 0.5–< 0.8 as medium, and 0.8 or above as large.^13^

## 3 RESULTS

### 3.1 Participants

Between April and June 2021, a total of 168 participants were consecutively enrolled in the study. All participants have not been diagnosed with COVID‐19. Two participants were excluded because they had received only one dose of the vaccine. Eleven were excluded from the analysis because routine tests, such as blood tests and temperature monitoring, were not performed on them. Two participants were excluded because we could not obtain their consent. Therefore, the study population consisted of 153 participants with a completed study. Among these 153 participants, 115 (75%) were female, six (4%) were obese (body mass index of at least 30 kg/m^2^), 58 (38%) were smokers, 30 (20%) had food allergies, and 38 (25%) had drug allergies (Table 1). A participant had experienced an episode of anaphylaxis in the past. The median age was 54 years (range 22–86), and 74 (48%) of the participants were over 55 years of age. Chronic diseases among participants were cardiovascular disease (*n* = 12, 8%), respiratory diseases (*n* = 14, 9%), chronic kidney disease (*n* = 10, 7%), autoimmune diseases (*n* = 1, 0.7%), thyroid disorders (*n* = 7, 5%), dyslipidemia (*n* = 58, 38%), hypertension (*n* = 41, 27%), diabetes mellitus (*n* = 14, 9%), and cancers (*n* = 5, 3%).

**Table 1:** Participant characteristics

Variables	*n* = 153
Age, years	52 (14)
Sex	
Female, *n* (%)	115 (75%)
BMI (kg/m^2^)	23 (4)
Body weight (kg)	59 (12)
BSA (m^2^)	1.6 (0.2)
Percent overweight (%)	4.2 (18.4)
Serum hemoglobin concentration (g/dl)	13.6 (1.2)
History of allergic diseases	
Perennial allergic rhinitis, *n* (%)	36 (24%)
Seasonal allergic rhinitis, *n* (%)	38 (25%)
Bronchial asthma	21 (14%)
Skin allergy	20 (13%)
Food allergy	30 (20%)
Drug allergy	38 (25%)

Abbreviations: BMI, body mass index; BSA, body surface area.

### 3.2 Postvaccination fever

Fever after receiving the COVID‐19 vaccine was reported more frequently by younger vaccine recipients (22–55 years old) than by older vaccine recipients (more than 55 years old) and more frequently after receiving the second dose than after the first dose. The frequency of fever (axillary temperature ≥37.3°C) after the first dose was 1.3% and after the second dose was 29.4%. During the 4‐day period, 41.8% of the younger vaccine recipients reported an axillary temperature of 37.3°C or higher after the second dose and 16.2% of the older recipients. None of the vaccine recipients reported high fever (axillary temperature, 38.9°C–40.0°C) after the first dose, compared to 1.3% after the second dose. No participant in the vaccine group reported temperatures of 40.0°C or higher. Twenty‐one (58%) of the participants with perennial allergic rhinitis (*n* = 36) had postvaccination fever (37.3°C or higher). Some participants (*n* = 20) had a slight fever between 37.0°C and 37.7°C 15 min after the second vaccination (axillary temperature [15 min]). Ten (50%) of these participants with mild fever had postvaccination fever. The fever occurred within 48 h after vaccination and resolved within 2 days after the onset of the fever. Vaccinated participants used acetaminophen more frequently after the second dose, compared to the first dose (12% after the first dose; 42% after the second dose; *p* < 0.01). Participants after the second vaccination represented significant differences in peak temperature with and without use of acetaminophen (37.3 [0.9]°C vs. 36.5 [0.6]°C; *p* < 0.01; Cohen's *d* = 1.08). Younger recipients were more likely to use acetaminophen for antipyretics or pain reliever (14% after first dose; 56% after second dose) than older recipients (9% after first dose; 28% after second dose).

### 3.3 Adverse events

There were no serious events such as anaphylaxis or deaths related to vaccination. No adverse event causing withdrawal from the study was reported. But allergic reactions without anaphylaxis (*n* = 4), including persistent cough, rash, and swelling of the throat, occurred after receiving the first dose of the COVID‐19 vaccine. All reported nonanaphylaxis allergic cases after receiving the vaccine occurred in women, not men. These cases underwent treatment with anti‐histamine (bilastine) in three (2%), corticosteroids (hydrocortisone sodium succinate) in one (0.7%) and fluticasone propionate/formoterol fumarate dehydrate in two (1%) (Table 1).

### 3.4 Analysis

Univariable regression analysis revealed that the peak axillary temperature after the second vaccination fever was negatively correlated with age, body weight, body surface area (BSA), percent overweight, or a history of lifestyle‐related disease (hypertension or dyslipidemia) (Table 2). Similarly, the peak temperature was positively correlated with the BSA to body weight ratio, a history of perennial allergic rhinitis, or axillary temperature (15 min). The peak temperature did not correlate with sex or other allergies.

**Table 2:** Univariable correlation between each parameter and peak axillary temperature after receipt of the second dose of the COVID‐19 vaccine

	Correlation coefficient	*p*
Age	−0.454	<0.001
Sex	0.003	NS
BMI	−0.129	NS
Body weight	−0.169	<0.05
BSA	−0.161	<0.05
Percent overweight	−0.182	<0.05
BSA to body weight ratio	0.246	<0.01
Axillary temperature (15 min)	0.234	<0.01
Serum hemoglobin concentration	0.143	<0.10
History of perennial allergic rhinitis	0.368	<0.001
History of seasonal allergic rhinitis	0.070	NS
History of bronchial asthma	0.092	NS
History of skin allergy	0.151	<0.10
History of food allergy	−0.021	NS
History of drug allergy	0.016	NS
Hypertension	−0.257	<0.01
Dyslipidemia	−0.229	<0.01

*Note*: Axillary temperature (15 min): axillary temperature 15 min after the second vaccination. Abbreviations: BMI, body mass index; BSA, body surface area.

**Figure 1:**
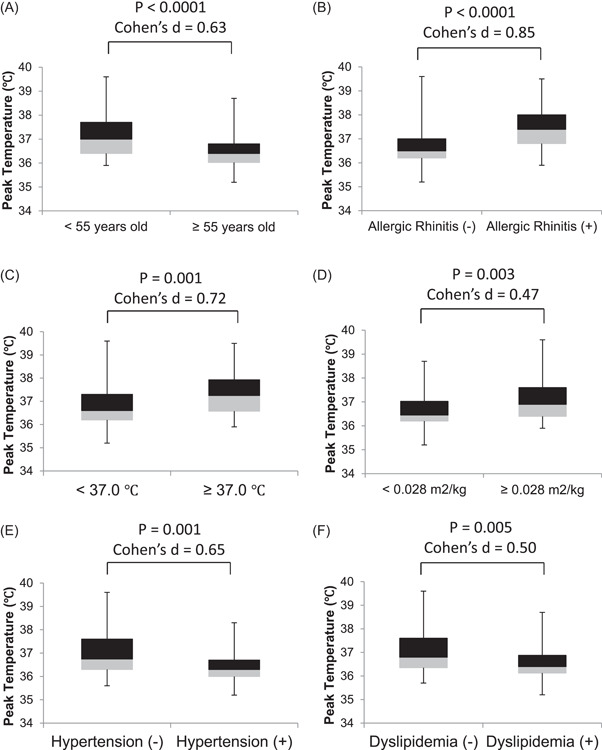
Comparison of the peak axillary temperature after the second vaccination in each group. (A) Younger (<55 years old; *n *= 79) versus older vaccine recipients (≥55 years old; *n *= 74), (B) recipients without (*n* = 117) versus with a history of perennial allergic rhinitis (*n* = 36), (C) recipients without (*n* = 133) versus with mild fever (*n* = 20) 15 min after vaccination, (D) recipients with a lower body surface area to body weight ratio (lower than 0.028 m^2^/kg; *n* = 88) versus more ratio (0.028 m^2^/kg or higher; *n* = 65), (E) recipients without (*n* = 112) versus with hypertension (*n* = 41), (F) recipients without (*n* = 95) versus with dyslipidemia (*n* = 58).

Each recipient is divided into a plurality of subgroups (Figure 1). As expected, the peak axillary temperature after the second vaccination in younger vaccine recipients (<55 years old; *n* = 79) was greater than in older recipients (≥55 years old; *n* = 74), with a medium effect size (37.1 [0.8]°C vs. 36.6 [0.8]°C; *p* < 0.0001; Cohen's *d* = 0.63) (Figure 1A). The peak temperature in recipients with perennial allergic rhinitis (*n* = 36) was also greater than in those without perennial allergic rhinitis (*n* = 117), with a large effect size (37.4 [0.9]°C vs. 36.7 [0.8]°C, *p* < 0.0001; Cohen's *d* = 0.85) (Figure 1B). The peak temperature in recipients with a slight fever of 37.0°C or higher 15 min after the second vaccination (*n* = 20) was also higher than in those without a slight fever (*n* = 133), with a medium effect size (37.4 [1.0]°C vs. 36.8 [0.8]°C, *p* = 0.001; Cohen's *d* = 0.72) (Figure 1C). The peak temperature in recipients with a higher ratio of BSA to body weight ratio (≥0.028 m^2^/kg; *n* = 65) was higher than in those with a lower ratio (<0.028 m^2^/kg; *n* = 88), with a small effect size (37.1 [0.9]°C vs. 36.7 [0.8]°C, *p* = 0.003; Cohen's *d* = 0.47) (Figure 1D). The peak temperature in recipients with hypertension (*n* = 41) was lower than in those without hypertension (*n* = 112), with a medium effect size (36.5 [0.7]°C vs. 37.0 [0.8]°C, *p* = 0.001; Cohen's *d* = 0.65) (Figure 1E). The peak temperature in recipients with dyslipidemia (*n* = 58) was lower than in those without dyslipidemia (*n* = 95), with a medium effect size (36.6 [0.8]°C vs. 37.0 [0.8]°C, *p* = 0.005; Cohen's *d* = 0.50) (Figure 1F).

Multivariable logistic regression analysis identified age (*p* < 0.0001), a history of perennial allergic rhinitis (*p* < 0.0001), BSA (*p* < 0.001), body weight (*p* < 0.001), percent overweight (*p* < 0.001), and serum hemoglobin concentration (*p* = 0.01) as independent predictors and the most powerful predictors of postvaccination fever (Table 3). The peak axillary temperature is the dependent variable (outcome) used in this regression model. A numerical model of human thermoregulation was derived from these specific data sets. The formula is as follows. Axillary temperature (*T*_Axilla_) = *b*_0_ + *b*_1_ × age + *b*_2_ × history of perennial allergic rhinitis + *b*_3_ × BSA + *b*_4_ × body weight + *b*_5_ × percent overweight + *b*_6_ × serum hemoglobin concentration.

**Table 3:** The independent predictors of postsecond vaccination fever selected in the logistic regression analysis

	Coefficient (*n* = 153)	OR	95% CI
Age (years)	−0.025	0.976	0.968–0.984
History of perennial allergic rhinitis	0.556	1.744	1.347–2.259
BSA (m^2^)	−14.113	7.424 × 10^−7^	6.762 × 10^−10^ – 8.150 × 10^−4^
Body weight (kg)	0.278	1.321	1.136–1.536
Percent overweight (%)	−0.077	0.926	0.887–0.965
Serum hemoglobin concentration (g/dl)	0.124	1.131	1.025–1.249

*Note*: The odds ratio is a measure of the increase in risk of postvaccination fever. Abbreviations: 95% CI, 95% confidence interval; BSA, body surface area; OR, odds ratio.

*T*_Axilla_ = 42.898 − 0.025 × age (years) + 0.556 × history of perennial allergic rhinitis (0 or 1) − 14.113 × BSA (m^2^) + 0.278 × body weight (kg) − 0.077 × percent overweight (%) + 0.124 × serum hemoglobin concentration (g/dl).

Sensitivity, specificity, and corresponding 95% confidential intervals (95% CI) of various cut‐off values are reported in Table 4. For an axillary temperature cut‐off value of 37.3°C, sensitivity was 66.1% (95% CI: 57.2%–72.6%) and specificity 90.7% (95% CI: 85.6%–94.5%). The multiple coefficient of determination (*R* ^2^) was 0.410.

**Table 4:** Sensitivity, specificity, PPV and NPV with 95% CI for detecting postsecond vaccination fever with axillary thermometers

Body temperature cut‐off	Sensitivity (95% CI)	Specificity (95% CI)	PPV	NPV
			(95% CI)	(95% CI)
37.0°C	0.754 (0.663–0.827)	0.844 (0.789–0.887)	0.741 (0.651–0.812)	0.853 (0.798–0.896)
37.3°C	0.661 (0.572–0.726)	0.907 (0.856–0.945)	0.804 (0.697–0.884)	0.822 (0.776–0.857)
37.5°C	0.351 (0.241–0.452)	0.914 (0.878–0.946)	0.565 (0.387–0.727)	0.815 (0.784–0.884)

Abbreviations: 95% CI,  95% confidence interval; NPV, negative predictive values; PPV, positive predictive values.

Fever occurred in a participant with Basedow's disease who received the COVID‐19 vaccine, but was unexpected in our model. The other six participants with thyroid disease had hypothyroidism or euthyroidism and did not have fever.

## 4 DISCUSSION

In this observational study, we searched for independent clinical and laboratory factors of postvaccination fever using a multivariable statistical approach. We found that age, a history of perennial allergic rhinitis, BSA, body weight, percent overweight, and serum hemoglobin concentration were the variables with the highest discrimination power for postsecond vaccination fever. Although the BSA to body weight ratio, a history of lifestyle‐related disease, or axillary temperature (15 min), were, using univariable analysis, associated with postvaccination fever, they provided no additional value in discriminating postvaccination fever.

This model identified younger age as a stronger predictor of postvaccination fever. Our result is consistent with the previous clinical trial that younger age was significantly associated with the frequency of fever after the second dose of the COVID‐19 vaccine.^7^ Lifestyle‐related diseases (hypertension or dyslipidemia) and aging are characterized by a state of chronic low‐grade inflammation,^14^, ^15^ so the immune system is constantly defending itself against chronic inflammation and does not respond well enough to combat an infectious agent. A state of chronic inflammation can weaken reactogenicity, such as postvaccination fever. Female gender was reported to be a regulatory factor for the immune response to COVID‐19 vaccination in the previous study,^16^ but this relationship was not observed in this study. The recipient with atopy history, such as asthma and eczema, was reported to have a severe systemic response (not fever) after the first dose of the influenza vaccine and a severe local response after the second dose.^17^ In fact, our results also showed that part of the allergy history, as well as younger age, has a strong impact on fever after vaccination, although parameters such as immunoglobulin E (IgE) or allergic tests were not performed. Intriguingly, the presence of allergic rhinitis can be associated with the infectivity of SARS‐Cov‐2 and the poor prognosis of COVID‐19, especially in medical personnel, because the incidence of allergic rhinitis is high with respect to occupational exposure, such as cleaning materials, body fluids, latex, and aerosol medications.^18^, ^19^ Improvement of allergic rhinitis can decrease the infectivity and severity of COVID‐19, because SARS‐Cov‐2 enters the epithelium of the upper respiratory tract. We recommend that healthcare workers with allergic rhinitis receive the COVID‐19 vaccination, although they may have postvaccination fever more frequently. Several studies have reported that a large BSA and a large body weight were beneficial in reducing body core temperature,^20^ and their formula^21^ was used in this model. In this formula, we substitute the percent overweight for the percent body fat. Other studies showed that elevation in rectal temperature was associated with increased serum hemoglobin under both control and heat exposure conditions.^22^ Serum hemoglobin, as well as hematocrit, are inverse indicators of blood volume, and people with concentrated blood can get dehydration fever. Although fever after vaccination did not correlate significantly with serum hemoglobin concentration (*p* < 0.10) in the univariable analysis, the association reached statistical significance (*p* < 0.05) in the multivariable analysis in our study. Additional considerations of morphological and hematological parameters to age and part of the allergy history as independent variables increased the precision of the prediction model for postvaccination fever. In our model, no fever participant was expected after vaccination with Basedow's disease. Thyroid hormone is a relevant factor in thermoregulatory control,^23^ and hyperthyroidism may be an additional factor in post‐second vaccination fever. No participant had been diagnosed with COVID‐19 before vaccination in this study. COVID‐19 infection before vaccination was reported to be associated with a serious adverse effect of the COVID‐19 vaccine in another study.^16^

Fever may have a beneficial role in the prevention of infection.^24^ The presence of postvaccination fever means that the immune system is building protection against invading pathogens, although high fevers can have detrimental effects on the host.^24^ Acetaminophen was previously reported to have led to a reduction in fever rates after vaccination.^25^ In the current study, prophylactic acetaminophen was not administered, but acetaminophen administered to participants for reasons other than fever (i.e., pain at the injection site) could possibly have masked some fever, making the observed fever rates less apparent. On the contrary, the peak temperature was actually higher in antipyretic vaccine recipients than those without antipyretics in another study,^26^ which had the same result as this study. Therefore, we thought that the peak temperature was less affected by acetaminophen. On the other hand, antipyretics, including acetaminophen and corticosteroids, have been suggested to inhibit antibody production due to the anti‐inflammatory effect, and antipyretic use is no longer recommended for vaccination‐associated fever in Canada and New Zealand.^27^ It is important to minimize the use of acetaminophen. Frequent intake of water and salt is recommended, although we should pay attention to the reduced cardiac and renal function of individuals with fever.

Regulation of body temperature is a fundamental homeostatic function in humans. This observational study highlights that younger age, part of the allergy history (perennial allergic rhinitis), morphological factors (small and light body), and a blood indicator of body water status (concentrated blood) are key regulators of a biological indicator of the inflammatory response to vaccination (high body temperature after vaccination). The peak temperature reported to us may be lower than the real temperature, as they do not want to go off work with suspected COVID‐19. This may reduce sensitivity in this study. We evaluated postvaccination fever in our population. However, this predictive model should be prospectively inspected in other population groups. Therefore, the purpose of the present study is not to continue its immediate use in practice, but to show a new tool for understanding vaccination‐associated fever. Through more fine‐tuning of the model that can predict vaccine‐associated fever, this approach offers useful information to vaccine recipients, which is likely to ease fever phobia.

### 4.1 Limitations of the study

Our single observational study has several limitations that should be considered when evaluating these results. First, the number of participants was relatively small for a definite conclusion to draw. More studies are clearly needed in a large number. Second, the rate of postvaccination fever can differ depending on the type of COVID‐19 vaccine, as previously described.^28^ However, other types of vaccines have not yet been administered in our hospital. Therefore, future studies could be required to investigate fever after the use of all authorized vaccines. Third, this was a single‐year study, and the viral antigen can vary from year to year. If the composition of the COVID‐19 vaccine changes according to new variants, fever rates after COVID‐19 vaccination could also vary depending on the composition of the vaccine. Fourth, all participants were Japanese in the current study. More countries and ethnic groups should be included in the further study. Fifth, the intensity of work can have a strong influence on body temperature.^20^ It is hoped that individual work conditions were added to the model. Sixth, this study was conducted in a cool climate. Because climate and clothing play an important role in body temperature,^18^ it may be necessary to add climate and clothing parameters to the model. Seventh, parameters such as IgE or allergic tests were not performed for the diagnosis of allergic rhinitis, although we interviewed participants about their symptoms and their history to diagnose them with allergic rhinitis. Part of perennial allergic rhinitis in this study may involve nonallergic rhinitis. Finally, we mention that future efforts to explore another contributing factor may be necessary to better understand postvaccination fever and improve the sensitivity of this model.

## 5 CONCLUSIONS

This study demonstrated that younger age, a history of perennial allergic rhinitis, a small and light body, and a higher serum hemoglobin concentration are independent predictors of postsecond vaccination fever. Although the predictive model is not validated for direct clinical use, it illustrates the clinical potential of the technique used. If people are more aware of these factors of postvaccination fever and the elements of concern are removed, vaccination will go smoothly. There is further discussion as to whether improving perennial allergic rhinitis and frequent intake of water and salt decrease the peak temperature after vaccination.

## AUTHOR CONTRIBUTIONS

**Satoru Chiba**: Conceptualization; formal analysis; investigation; methodology; project administration; supervision; validation; visualization; writing—original draft; writing—review and editing. **Kaoru Shinohara**: Project administration; supervision.

## CONFLICT OF INTEREST

The authors declare no conflict of interest.

## TRANSPARENCY STATEMENT

The lead author affirms that this manuscript is an honest, accurate, and transparent account of the study being reported; that no important aspects of the study have been omitted; and that any discrepancies from the study as planned (and, if relevant, registered) have been explained.

## ETHICS STATEMENT

This study was carried out according to the Declaration of Helsinki guidelines and was approved by the Ethics Committee of the Sapporo Suzuki Hospital (No. 2021‐001, April 15, 2021) and the procedures were in accordance with institutional guidelines. Written informed consent was obtained from each study participant. We also obtained permission to publish our research results in writing. Before analyzing the data, all identifiable information was deleted and specific individuals cannot be identified by these data. Therefore, this was approved by the ethics committee.
